# Coproduction of lipids and carotenoids by the novel green alga *Coelastrella* sp. depending on cultivation conditions

**DOI:** 10.1016/j.btre.2022.e00769

**Published:** 2022-10-24

**Authors:** Mizuki Saito, Haruka Watanabe, Mitsuki Sasaki, Madoka Ookubo, Takashi Yarita, Masakazu Shiraiwa, Munehiko Asayama

**Affiliations:** a College of Agriculture, Ibaraki University, 3-21-1 Ami, Ibaraki 300-0332, Japan; b United Graduate School of Agricultural Science, Tokyo University of Agriculture and Technology, 3-5-8 Fuchu, Tokyo 183-8509, Japan

**Keywords:** Biorefinery, Carotenoid, Coelastrella, Dual production, Lipid

## Abstract

A novel green alga *Coelastrella* sp. D3–1 was isolated, and its unique and significant lipid and carotenoid coproduction capability was characterised depending on cultivation conditions. The main component of produced lipids was triacylglycerol under nutrient depletion conditions, in which fatty-methyl-esters made up 20−44% of the dry cell weight (DCW) and consisted of abundant C16:0 and C18:1 fatty acids. The red (orange)-stage cells also produced a large portion of carotenoids (38.5% of the DCW) involving echinenone, canthaxanthin, and astaxanthin as major components accumulated over only 5–6 days under optimal conditions. Stress tests revealed resistance of the cells to pH 2–11, high temperatures (40–60 °C), ultraviolet irradiation, drought, and H_2_O_2_ treatment, thereby showing a robust nature. Both green- and red (orange)-stage cell extracts also showed antioxidant and anti-inflammatory abilities, implying that they have significant functions as useful biorefinery materials.

## Highlights

• Lipid/carotenoid production by *Coelastrella* sp. D3-1 was characterised.
• Triacylglycerol, echinenone, canthaxanthin, and astaxanthin were found at red stage.
• Cells were resistant to pH 211, 4060 °C, UV irradiation, drought, and H_2_O_2_.
• Cell extracts showed antioxidant and anti-inflammatory properties.
• *Coelastrella* sp. D3-1 and the derivatives are useful biorefinery materials.

## 1 Introduction

Microalgae are model microorganisms that have effective photosynthetic capabilities and are used as a resource in biorefineries to produce fuels, fertilisers, feed, fiber, and food [1,2], culture systems can use economic media and may not be affected by season and location. Some green algae (e.g. *Chlorella, Nannochloropsis, Nephroselmis*, and *Chlamydomonas*) have been used to produce triacylglycerol (TAG), a neutral lipid that can be used as a biodiesel or food oil [3], [4], [5], [6], [7], [8]. In such cases, the properties of the oil depend on the structure determined by the number of carbons and position/number of double bonds. Therefore, the productivity of C16/C18 fatty acid derivatives in cells could be important when oil production is examined in biorefineries. In addition, green algae, such as *Haematococcus* and *Dunaliella*, are good producers of β-carotenoids, such as astaxanthin (Ax), depending on the specific cultivation conditions under nutrient depletion or high salt concentration, respectively, which allows the red pigments to accumulate in the cells without high-level of lipid production [9]. The production of β-carotenoids has also been reported in non-photosynthesising oceanic microorganisms, such as *Paracoccus* and *Labyrinthulea,* or genetically modified *Escherichia coli*, cyanobacteria, and plants [10], [11], [12]. In these, the key enzyme CtrW/CtrZ may catalyze the conversion of β-carotene (βCar) to echinenone (Ec)/canthaxanthin (Cx) and Ax.

Regarding the coproduction of lipids and β-carotenoids, although the green algae *Coelastrella* is a good producer of biorefinery materials, productivity depends on strains and cultivation conditions, such as temperature (15−25 °C), light intensity, CO_2_ supply, and medium composition [13], [14], [15], [16]. For high coproduction of lipids and β-carotenoids from *Coelastrella* under optimal conditions, there is a need to obtain strains that can grow at temperatures of 30 °C or higher and to establish highly efficient culture methods using strains that are resistant to environmental stresses.

In this study, a novel and robust green alga, *Coelastrella* sp. strain D3–1, was successfully isolated and characterised as a dual producer of specific oils (lipids) and fat-soluble pigments, such as β-carotenoids and chlorophylls, obtained under different culture conditions at 30 °C. Both functional materials accumulated in the earliest period at significant levels were verified and compared to those of other *Coelastrella* cases reported to date. It was also examined for the first time that the cells could tolerate environmental stress, which could be applied to biorefineries, involving practical large-scale outdoor and indoor cultivation systems. The green/red-cell extracts showed functions antioxidant and anti-inflammatory functions. From these findings, the impacts of the usefulness of this strain and its products are discussed as biorefinery materials.

## 2 Materials and methods

### 2.1 *Isolation of Coelastrella strains*

*Coelastrella* sp. D3–1 and other *Coelastrella* strains were isolated from an outdoor cell culture consisting of natural water collected from an area in Kanagawa, Japan, using a mixed and diluted overlay method on BG11 agar plates at 30 °C [17,18]

### 2.2 *Identification of the D3–1 strain*

**Fig. 1:**
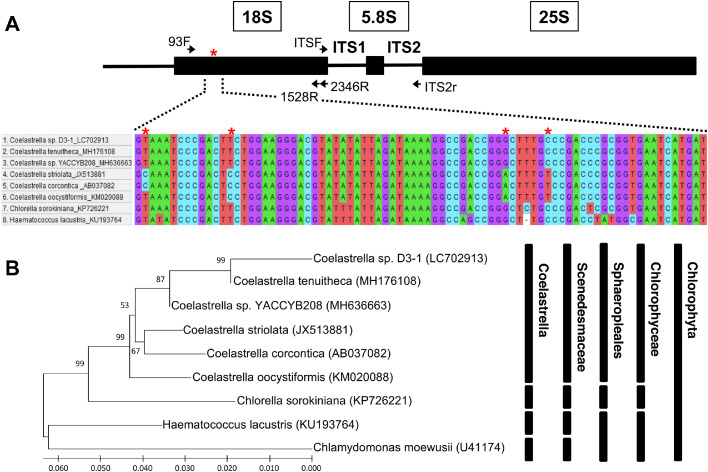
Identification of isolated *Coelastrella* sp. D3–1. (A) The 18S rDNA–ITS1–5.8S–ITS2 region on the genome and the nucleotide sequence alignment of a part of the region. The region (3,258 bp, DDBJ accession number LC702913) was amplified via PCR with a set of primers (93F and ITS2_r), cloned, sequenced, and subjected to database analysis. Asterisks show distinct nucleotide sequences between D3–1 and other *Coelastrella* sp. strains. (B) Phylogenetic analysis of the 18S rDNA sequences was used for the classification of D3–1 cells. Respective cases are shown with accession numbers. A bootstrap test was performed with 1,000 replicates. The 18S rDNA sequence of *Chlamydomonas moewusii* was utilised as an out-group. The scale bar indicates 〜6% sequence divergence.

The 18S rDNA of the D3–1 strain was amplified via PCR using primers 93F (5′- CTGCGAATGGCTCATTAAAWCAG-3′) and ITS2_r (5′- TCCTCCGCTTATTGATATGC-3′) (Fig. 1). The amplified 3.3-kilobase pairs (kbp) DNA fragment was cloned into the cloning vector pGEM-T to produce pD3–18S. The 3,258-bp nucleotide sequence (DDBJ accession number LC702913) of the 18S rDNA–ITS1–5.8S–ITS2 region was verified using the dideoxy method with the universal primers 93F (5′-CTGCGAATGGCTCATTAAAWCAG-3′), 1528R (5′-CCTCTAGGTGGGAGGGTTTAATG-3′), 2346R (5′-GMAACCTTGTTACGACTT-3′), ITSF (5′-AAGTCGTAACAAGGTTTCCG-3′), and ITS2_r. The 2.5-kbp (or 1.7-kbp) 18S rDNA sequences from the respective strains were aligned using CLUSTAL W and integrated into the MEGA10 package from MEGA6 [20] (Fig. 1A), for phylogenetic analysis and cell identification [18,21] (Fig. 1B).

### 2.3 Cultivation conditions

#### 2.3.1 Standard cultivation conditions (SCC)

Cells were cultured under static (standing) conditions with 0.04% CO_2_-containing air in 50 mL BG11 or CB medium [22] in a 100 mL Erlenmeyer flask, in which pre-cultured cells (0.5 mL) were inoculated at 30 °C. Alternatively, they were cultured on BG11 or 0.2BG11 (BG11 medium diluted 1:4 with water) plates for several months under light irradiation of 30 or 100 μmol photons *m*^−2^ *s*^−1^ at 30 °C to stimulate the accumulation of lipids and pigments. The ISL device (CCS Co Ltd., Kyoto, Japan; mini, 150 × 150 mm, F/R) was used for LED irradiation when needed. During cultivation, a part of the culture or cells scraped from the plate was sequentially harvested, and cell turbidity was measured at OD_730_ using MULTISKAN GO (Thermo Scientific Co. Ltd., Tokyo, Japan) [17]. Microscopic cell observations were also performed when needed.

#### 2.3.2 Basal induction conditions (BIC)

Cells were pre-cultivated in 50 mL BG11 in a 100 mL Erlenmeyer flask at 30 °C for 5 days. The flasks were placed in a CF-415 cultivation chamber (TOMY Co., Ltd., Tokyo, Japan) with a 2% CO_2_ supply and were subjected to continuous reciprocating shaking (40 rpm) under white light conditions at 100 μmol photons *m*^−2^ *s*^−1^ at 30 °C. The ISL device (CCS Co Ltd., Kyoto, Japan; big, 305 × 302 mm, F/R/G/B) was used for LED irradiation. Five (or ten) mL of the cell culture was collected, and the cells were suspended in 50 mL of fresh BG11 or BG11–P medium (phosphorous-deficient medium without K_2_HPO_4_) in 300 (or 500) mL Erlenmeyer flasks. The flasks were placed at 30 °C in the same cultivation chamber to induce the production of lipids and pigments. For lipid or pigment extraction, cells were collected on the 5^th^ or appropriate day during the main cultivation.

### 2.4 Microscopic observation

An aliquot of the cell culture or cells scraped from the plate was observed under an optical microscope (BX53: Olympus, Tokyo, Japan) under differential interference contrast conditions with a shutter speed of 0.48 s [18].

### 2.5 Lipid content detection

**Fig. 2:**
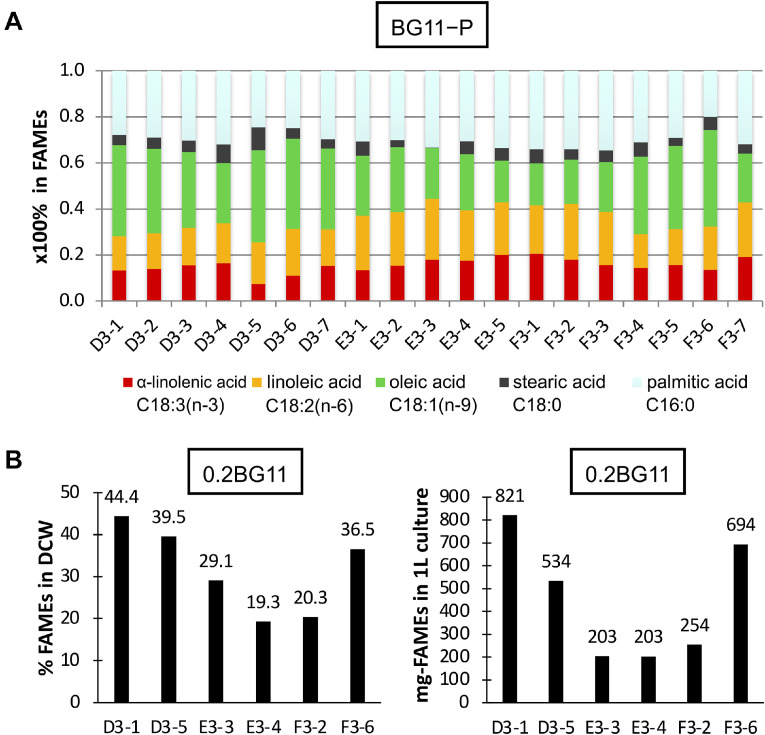
Lipid production in isolated green algae. (A) A total of 19 isolated green algae were cultivated in BG11–P medium under a static condition with white-light irradiation (30 μmol photons *m*^−2^*s*^−1^) in 0.04% CO_2_ air at 30 °C for 14 days. FAMEs derived from the cells were subjected to FID analysis and methyl-esterified fatty acid compositions were determined as a percentage (%). (B) The six strains were cultivated in 0.2BG11 under a reciprocating shaking condition (40 rpm), with white-light irradiation (100 μmol photons *m*^−2^*s*^−1^ in an incubator with 3% CO_2_-containing air at 30 °C for 6 days. Total lipids (fatty acids) were extracted from the cells and FAMEs were subjected to FID analysis. Total amounts of FAMEs from DCW or 1 litter of cell culture are shown as a percentage (%) in the left panel or as mg in the right panel, respectively.

A total of 19 isolated green algae were cultivated in 50 mL of BG11–P medium in an Erlenmeyer flask (100 mL) under a static condition with white-light irradiation (30 μmol photons *m*^−2^ *s*^−1^) in 0.04% CO_2_-containing air at 30 °C for 14 days. The cells were harvested, dried, and total lipids (fatty acids) were extracted [18] (Fig. 2A). The six strains were cultivated in 50 mL of 0.2BG11 medium in an Erlenmeyer flask (300 mL) and subjected to reciprocating shaking (40 rpm) under white-light irradiation (100 μmol photons *m*^−2^ *s*^−1^) in an incubator (CF-415: TOMY Ltd., Tokyo, Japan) with 3% CO_2_ at 30 °C for 6 days (Fig. 2B). Lipids were also extracted from cells incubated with Eicosan as an internal standard, and lipids were converted to fatty acyl methyl esters (FAMEs) [18] and subjected to gas chromatography/flame ionization detection (GC/FID) as described previously [23]. The amount of accumulated FAME was expressed per DCW or per 1 L of cell culture.

### 2.6 Preparation of green or red extracts containing lipids and pigments

The green- or red-stage D3–1 cells (50 mL culture) grown under SCC for 1 or 3 months were harvested and freeze-dried using a freeze dryer (FZ-Compact: Labconco Co. Ltd., Kansas City, USA). The DCW was measured, and the samples were subjected to cell disruption with glass beads (φ = 1 ± 0.1 mm) and a mixture of diethyl ether:chloroform:methanol (400 μL, 1:2:1) using Vortex-Genie 2 (Scientific Industries, Inc., New York, USA). The supernatants were obtained via centrifugation (8,000 × *g,* 10 min). This extraction was repeated four times and approximately 1.6 mL of the extract was prepared. Notably, the cell disruption rate was measured using an optical microscope. These rates were considered to calculate the yield of the extracts. The resultant samples were subjected to a rotary evaporator (V-850; BUCHI, Flawil, Switzerland), and the dried extracts were weighed (approximately 10−30 mg per sample) and stored at −30 °C until use.

### 2.7 Pigment or lipid analysis via thin-layer chromatography (TLC)

**Fig. 3:**
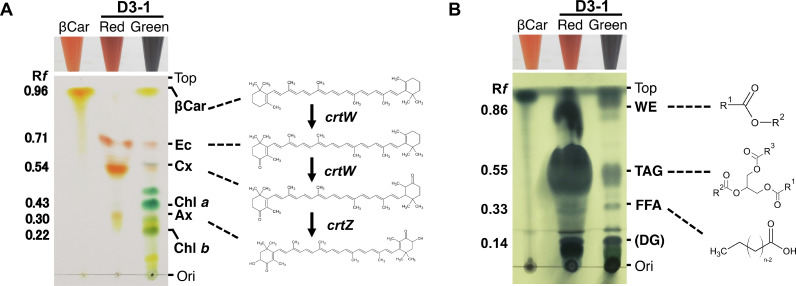
TLC analysis for pigments and lipids. TLC analysis was performed for pigments (A) or FAMEs (B). A 20 μL aliquot of the extract was spotted on the origin (ori) of a silica gel plate. This plate was subjected to TLC with a developer eluent. Positions referring to respective pigments are shown as β-carotene (βCar), echinenone (Ec), canthaxanthin (Cx), astaxanthin (Ax), and chlorophylls on the right. The R*f* values are shown on the left. Chemical structures of respective compounds are also shown with biosynthesis genes.

TLC analysis of the fat-soluble β-carotenoid and chlorophyll pigments accumulating in cells that simultaneously produced lipids was performed as described previously with some modifications [18]. The dried extracts described in the previous section were dissolved in 1 mL of diethyl ether, and a 20 μL aliquot of each sample was spotted onto a silica gel plate (#60F254; Merck Millipore, Darmstadt, Germany). The pigments were developed using petroleum ether and acetone mixture as the eluent at a 4:1 ratio (Fig. 3A). Commercially available βCar (CAS RN 7235–40–7: Wako Co. Ltd., Osaka, Japan), Ax (CAS RN 472–61–7: DHI institute/Wako Co. Ltd.), Cx (CAS RN 514–78–3: CaroteNature/Wako Co. Ltd.), Ec (CAS RN 432–68–8 DHI institute/Wako Co. Ltd.), and Chl *a* (CAS RN 479–61–8: Fuji film Co. Ltd/Wako Co. Ltd.) were purchased. The band signal intensities corresponding to the respective pigments on the TLC gel were analysed using an imaging analyser (BIO-1D system: Vilber Lourmat Co. Ltd., Cedex, France) and expressed as% (w/w) of the total oil-soluble pigments of the extracts.

To visualize lipids on a silica gel plate, a 20 μL aliquot of the extract was also spotted on the origin (ori) of the plate (Fig. 3B). This plate was subjected to TLC, developed using hexane:diethyl ether:acetic acid mixture as the eluent at a ratio of 80:20:1 and stained with 5% (v/w in EtOH) phosphomolybdic acid solution (#32,186–00: Kanto Kagaku Co., Ltd., Tokyo, Japan) [18].

### 2.8 Pigment analysis via high-performance liquid chromatography (HPLC)

Pigments were identified via HPLC [19]. Ten microliters of the sample extract or standard prepared to the appropriate concentration with ethanol were applied to a Chromaster HPLC system (Hitachi High-Tech, Tokyo, Japan) with a Develosil C30-UG-5 column (3.0 mm i.d. × 250 mm, Nomura Chemical, Seto, Japan). In this analysis, the separation was performed at 25 °C and the absorbance was detected at 450 nm. The mobile phases were premixed with buffer A (91% methanol [MeOH], 5% methyl tert‑butyl ether (MTBE), 3.9% H_2_O, and 0.1% formic acid) and buffer B (46% MeOH, 50% MTBE, 3.9% H_2_O, and 0.1% formic acid). The total flow was 0.425 mL min^−1^, and the following gradient was applied: 0–5 min, 1% of buffer B, 5–55 min, 100% of buffer B, 55–75 min 100% of buffer B. Three minutes later, from 78 to 82 min, the gradient was set back to 1% of buffer B to complete one set of the analysis. Separated peaks were identified and each peak area was quantified. Of note, at this time, the calculation of the composition ratio of pigment production was evaluated using the average of TLC and HPLC values (Tables S3 & 1).

**Table 1:** *Coelastrella* sp. strains producing lipids and carotenoids.

TAP, Tris-acetate-phosphate; BBM, Bold's basal medium; B3N, Bold's triple nitrogen medium; W/R_LED, white + Red LED irradiation; ND, not determined; BIC, basal induction conditions; SCC, standard cultivation conditions.

Ax, astaxanthin; βCal, β-carotene; Cx, canthaxanthin; Ec, echinenone; Lt, lutein; Pf, phytofluene; Vx, violaxanthin; Chl*a*, chlorophyll *a*.

The values as% in five (βCal, Ec, Cx, Ax, Chl*a*) kinds of pigments are measured from TLC and HPLC analyses and shown as average values in the D3–1 case.

### 2.9 Spot assay for stress resistance

**Fig. 4:**
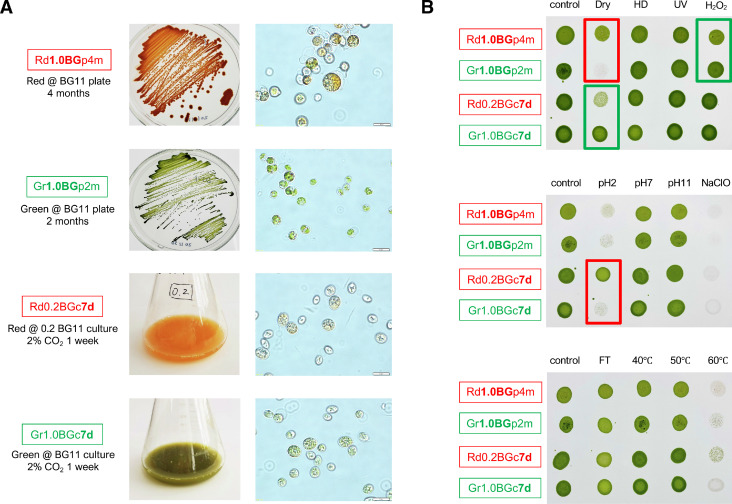
Red/green-stage cells and their stress resistance. (A) D3–1 cells were cultivated under four distinct SCC (Rd1.0BGp4m, Gr1.0BGp2m, Rd0.2BGc7d, and Gr1.0BGc7d), harvested, and exposed to several stresses. The appearance of cultured cells and the optical micrograph of the red/green-stage cells are shown. Scale bar: 10 μm. (B) After the stress treatment, the same amounts of aliquots of resultant cells were spotted on BG11 plates and the cells were grown under SCC for 7 days. The stress items and prepared cell types are shown. If there is a difference in growth, it is indicated by a square frame.

*Coelastrella* sp. D3–1 cells were cultivated at 30 °C on BG11 plates under SCC for samples Rd1.0BGp4m (Red/BG11/plate/4 months) or Gr1.0BGp2m (Green/BG11/plate/2 months), in which cells were grown on the same medium but at different cultivation periods. In contrast, the cells were cultivated at 30 °C in the 0.2BG11/BG11 liquid culture under BIC for samples Rd0.2BGc7d (Red/0.2BG11/culture/7 days) or Gr1.0BGc7d (Green/BG11culture/7 days), in which cells were grown for the same period but in different BG11 media. The cells were then collected and exposed to the respective stress conditions described previously [18], with modifications (Fig. 4). For heat stress, 100 μL of the cell culture was harvested, transferred into a 1.5 mL microtube, and then exposed to 40, 50, or 60 °C for 5 min. For pH stress, 100 μL of the cell culture was harvested and transferred into a 1.5 mL microtube. A 10 μL aliquot of 1 N hydrochloric acid (cell culture at pH 2) or 0.5 N sodium hydroxide (cell culture at pH 11) was added to the culture. For pH 7, the cell culture was directly spotted onto a BG11 agar plate under the same conditions as the control sample. For heat-dry stress, 100 μL of the cell culture was harvested, and the cell pellet was collected via centrifugation at 15,000 × *g* and subsequently exposed to 42 °C for 3 h in a dryer 2–2195 (ISUZU Seisakusho Co. Ltd., Tokyo, Japan). Next, 100 μL of fresh BG11 medium was added to the dried cell pellet and mixed thoroughly. For vacuum dry stress, 100 μL of the cell culture was harvested via centrifugation and the cell pellet was subjected to a decompression dryer TSW-100 (IKEDA Scientific Co. Ltd., Tokyo, Japan) for 5 min at room temperature. For freeze and thaw stress, 100 μL of the cell culture was kept at −80 °C until use. The samples were thawed at room temperature. For ultraviolet (UV) stress, 100 μL of the cell culture was harvested, transferred to a 96-well plate, and UV irradiated (100 μmol photons *m*^−2^ *s*^−1^, with 260-nm light) for 5 min at a distance of 8 cm. For sodium hypochlorite (NaClO) stress, 100 μL of the cell culture was harvested via centrifugation, and 100 μL of 300 parts per million (ppm) of sodium hypochlorite was added to the cell pellet for 3 min. The cells were collected via centrifugation once more, and fresh BG11 medium (100 μL) was added to the cell pellet and mixed well. For hydrogen peroxide (H₂O₂) stress, 100 μL of the cell culture was harvested and the cell pellet was mixed with 100 μL of 10 ppm hydrogen peroxide. After these stress treatments, 5 μL aliquots of the cell culture were spotted on BG11 agar plates. The control indicated that cells were spotted without stress. These cells were incubated under 0.04% CO_2_-containing air at 30 μmol photons *m*^−2^ *s*^−1^ of white fluorescent light at 30 °C for 7 days.

### 2.10 Antioxidant capacity measurement

**Fig. 5:**
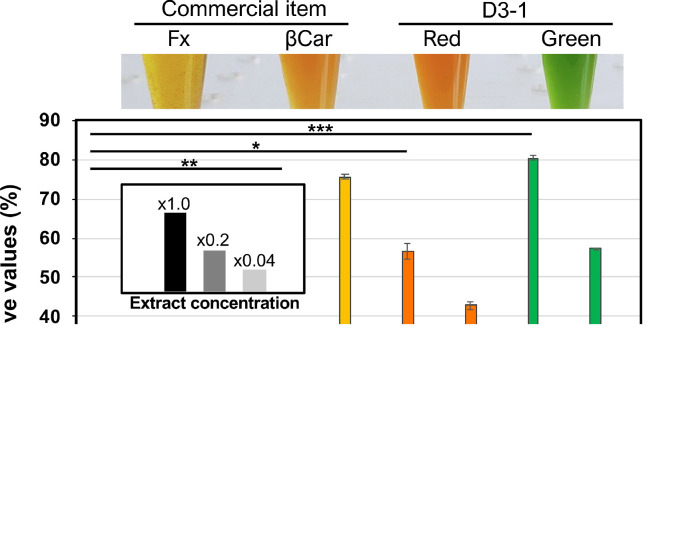
Antioxidant capacities of D3–1 red/green extracts. Antioxidant capacities were measured using the ABTS method. 20 μL aliquots of sample extracts under SCC at concentrations of × 1.0 (high), × 0.2 (medium), × 0.04 (low) were prepared and mixed with 180 μL of ABTS working solution, and the absorbance (A_734_) of the mixture was measured. Fx, fucoxanthin; βCar, β-carotene. The maximum antioxidant capacity was taken as 100% and the relative values are shown. The sample extracts (× 1.0 concentration) in microtubes are shown at the top. The measurements were performed according to three independent experiments and error bars are also shown. Significance was determined using a *t*-test: *, *P* < 0.01; **, *P* < 0.00001; ***, *P* < 0.0000001.

The green or red extracts from SCC dried using an evaporator were dissolved in an appropriate amount of ethanol as the × 1 original sample, resulting in final concentrations of 10 μg/μL of Fx, 1 μg/μL of βCar, 53 μg/μL of D3–1_red, and 21 μg/μL of D3–1_green. The resultant solutions were also diluted 5 (× 0.2) and 25 (× 0.04) folds with ethanol. The antioxidant capacity was measured using the 2,2′-azino-bis *(3)*-ethylbenzothiazoline-6-sulfonic acid (ABTS) method [24,25] (Fig. 5). An ABTS stock solution was prepared by mixing equal amounts of ABTS (7 mM) and potassium persulfate (2.45 mM). After storage in the dark for 18 h, the stock solution was diluted with phosphate-buffered saline (PBS; 20 mM, pH 7.4) to make a working solution with an absorbance of 0.70 ± 0.05 at 734 nm. To measure the antioxidant potential, a 20 μL aliquots of sample extracts at concentrations of × 1.0 (high), × 0.2 (medium), and × 0.04 (low) were prepared and mixed with 180 μL of ABTS working solution, and the absorbance (A_734_) of the mixture was measured. Fucoxanthin (Fx: 200, 40, 8 μg/20 μL), β-carotene (βCar: 20, 4, 0.8 μg/20 μL), D3–1_red extract (D3–1_red: 1,060, 212, 42.4 μg/20 μL), and D3–1_green extract (D3–1_green: 420, 84, 16.8 μg/20 μL) were used. Ethanol was used as the negative control. Absorbance at 734 nm was measured after incubation for 5 min at room temperature in the dark. ABTS radical scavenging activity (S%, antioxidant ability) was determined using the following equation:

$$S\%=\frac{A\mathrm{cont}\mathrm{rol}-A\mathrm{test}}{A\mathrm{cont}\mathrm{rol}}\;\times \;100$$

Where A_control_ is the absorbance of a blank control (a mixture of ABTS working solution and ethanol) and A_test_ is the absorbance of ABTS operating on the sample solution. Commercially available fucoxanthin (Fx, CAS RN 3351–86–8: DHI institute/Wako Co. Ltd.) and β-carotene (βCar) were purchased, respectively.

### 2.11 Anti-inflammatory analysis for red/green extracts

**Fig. 6:**
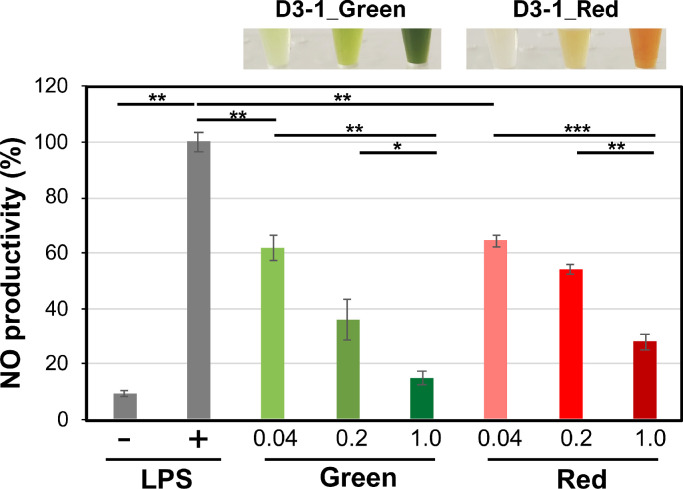
Anti-inflammatory properties of D3–1 red/green extracts. Anti-inflammatory properties of the extracts were measured using the Griess method. A 10 μL aliquot of extract under SCC at different concentrations (× 1.0, × 0.2, × 0.04) was prepared and subjected to 100 μL cell culture of the mouse macrophage RAW264 with LPS (10 μL). LPS was used as a control for nitric monoxide (NO) production (+, with LPS; −, without LPS). NO production with LPS is relatively shown as 100%. Sample photography is shown at the top. The measurements were performed according to three independent experiments and error bars are also shown. Significance was determined using a *t*-test: *, *P* < 0.075; **, *P* < 0.05; ***, *P* < 0.001.

Anti-inflammatory analysis of the extracts was performed using a previously described method [26], with modifications (Fig. 6). The freeze-dried green- (13 mg) or red (35 mg)-stage extracts from D3–1 cells from SCC were diluted with 240 μL of dimethyl sulfoxide (DMSO). Aliquots of the originals (120 μL) were diluted with 880 μL of PBS as the × 1 extract (green, 6.5 μg/μL; red, 17.5 μg/μL). These extracts were also diluted with PBS to obtain × 0.2 (green, 1.30 μg/μL; red, 3.50 μg/μL) or × 0.04 (green, 0.26 μg/μL; red, 0.70 μg/μL) solutions. These extracts (10 μL) were added to 100 μL of mouse macrophage RAW264 cells (1.0 × 10^5^ cells/well, RIKEN Cell Bank, Saitama, Japan) with the addition of lipopolysaccharide (LPS, a final concentration of 50 ng/mL) in a 96-well plate. The resultant culture was placed in an incubator (Steri-Cycle CO_2_; Thermo Fisher Scientific Inc., Tokyo, Japan) and cultivated with 5% CO_2_ at 37 °C for 12 h. Then, 50 μL of each culture was harvested and transferred to a new 96-well plate. The resulting products were added to 50 μL of sulfanilamide (Griess Reagent System; Promega, Tokyo, Japan), shaded, and reacted for 8 min. Next, 50 μL of the NED (n-1-naphylethylenediamine dihydrochloride) solution (Griess Reagent System) was added to the samples, which were then shaded and allowed to react for 8 min. Nitric monooxide (NO) absorbance was measured at 540 nm using a Flex Station 3 spectrometer (Molecular Devices Co. Ltd., Tokyo, Japan). The 50% inhibitory concentration (IC50) was calculated from triplicate experiments.

### 2.12 Statistical analysis

Statistical analysis was performed using a *t*-test [27]. It was used to calculate the *p*-value and to compare the differences in the antioxidant and anti-inflammatory properties of each extract. Differences were considered statistically significant at *p* < 0.05.

## 3 Results and discussion

### 3.1 Isolation and identification of D3–1

Aliquots of samples from an aqueous area in Kanagawa, Japan, were harvested, and 19 types of algal cells were isolated on BG11 plates. The nuclear 18S rDNA gene and ITS regions (Fig. 1A) of the D3–1 isolate were sequenced and compared to those (18S rDNAs as 2.5, 2.2, or 1.7 kbp) of similar species in a database (Fig. 1B). The 2,524 bp of D3–1 18S rDNA revealed several distinct nucleotide sequences within the 89–168 region (Fig. 1A) compared to those in other *Coelastrella* species. The D3–1 18S rDNA possessed 333 or 315 bp of distinct nucleotide sequences and showed 87.3% and 87.9% similarity to the full-length region of *Coelastrella oocystiformis* (KM020088 for accession number of 18S rDNA) and *Coelastrella corcontica* (AB037082), respectively. In contrast, the other two *Coelastrella tenuitheca* (MH176108) and YACCYB208 (MH636663) strains showed short sequences of 18S rDNA as 1,657 and 1,673 bp, respectively, indicating structural differences of 18S rDNA between two groups of D3–1/*oocystiformis*/*corcontica* and *tenuitheca*/YACCYB208. Furthermore, the 18S rDNA sequence of *Coerastrella striolata* (JX513881) was also short as 2,221 bp, which was a different size compared to that of the D3–1 strain. These results suggested that the D3–1 strain belonged to a different strain from the originating *Coelastrella striolata* strain. Therefore, this strain was designated as the green alga *Coelastrella* sp. D3–1, a novel strain belonging to the genus *Coelastrella*, family Scenedesmaceae, order Sphaeropleales, class Chlorophyceae, and phylum Chlorophyta.

### 3.2 Lipid production in isolated strains

Microalgae can efficiently fix CO_2_, resulting in the production of useful organic matter. Green algae are good producers of TAG as a source of biodiesel or food additives [26,[28], [29], [30], [31], [32], [33], [34], [35], [36], [37]]. Although *Coelastrella* can also produce useful lipids and pigments, there is insufficient knowledge of their productivities and properties, depending on cultivation conditions. In the following experiments, the incubation temperature was set at 30 °C, which was expected to increase the yield of lipid and pigment production in a short period, because *Coelastrella* has been previously tested at temperatures between 15 and 25 °C. In contrast, the use of media depleted of phosphorus or nitrogen sources is effective in the efficient accumulation of lipids in green algal cells [[4], [5], [6], [7],^18^]. Therefore, the isolated 19 strains were cultivated in BG11−*P* medium at 30 °C, and the results are shown in Fig. 2A. The isolated strains produced FAMEs (C16:0 and C18:1) at a percentage of more than 60% of the total FAMEs under BG11−*P*. Of these, six were selected from the D, E, and F strains as an efficient production stock. Using those promising strains, we further tested lipid induction using 0.2BG11 (diluted five folds BG11 with water) medium. This medium is considered to be significant for the coproduction of lipids and pigments because it is cost-effective and does not require bacterial collection from the culture before the induction, and it is also consistent with the conditions for pigment induction, as described later in this manuscript. The result showed that the D3–1 or F3–6 strain efficiently produced lipids (FAMEs) at 44.4 or 36.5% per DCW and 821 or 694 mg per 1 L culture under 3% CO_2_ after 6 days of cultivation (Fig. 2B). This indicated that the significant lipid productivities were 136.8 or 115.6 mg *L*^−1^ *d*^−1^, respectively. Therefore, we used D3–1 (and F3–6) for subsequent experiments.

The culture conditions were further verified for the combination of culture medium and flask volume. The results of this study were very important because these factors are directly related to the incorporation of the CO_2_ supply into the culture medium under stirring conditions and efficient biomass production in a short term. The results are shown in Supplementary Fig.e S1. When the cells were cultivated under several different conditions, the cell culture color interestingly changed to orange at the red (orange) stage, in which cells could effectively accumulate lipids (Fig. S1B). Especially, case C (5 + 50 mL of culture in 300 mL flask) suggested a good balance of lipid accumulation, at approximately 20−44% of DCW (Figs. 2 and S1, Table S1). This lipid accumulation was significantly higher than that observed in other *Coelastrella* sp. strains (Table 1) and other green algae [13], [14], [15], [16], indicating that 0.2BG11 was effective for D3–1 lipid production in only 5 days. Based on the results of this test, we named this condition BIC and also used it in pigment induction described later in this manuscript, comparing it with SCC.

We also inspected the lipid composition using TLC, which showed that TAG was the major product at more than 80% in total lipids (Fig. 3). Case C (Fig. S1) indicated that C16:0 (29.8%) and C18:1 (37.1%) were mainly produced by D3–1 cells among the total FAMEs (Table S1), showing a unique property of *Coelastrella* sp. cells reported to date (Table 1). This high accumulation of C16:0 and C18:1 lipids may indicate their use as food additives rather than biofuels [18].

### 3.3 Pigment production under different liquid culture conditions

The green alga *Coelastrella* sp. is also known to produce β-carotenoids. Therefore, we also inspected the ability of D3–1 to produce pigments, depending on culture conditions, which involved changing experimental conditions like CO_2_ supply (0.04 or 2%), light intensity (white or red), shaking or standing cultivation, and type of medium (BG11 or 0.2BG11) under BIC or SCC (Supplementary Fig. S2). When the cells were cultivated under liquid BIC, cell turbidity and β-carotenoid production were more effective than those under liquid SCC, indicating that high CO_2_ concentration and stirring are important. Although the BG11 medium was excellent for effective biomass and β-carotenoid production, 0.2BG11 medium could be used for a short period of β-carotenoid production even after 6–9 days at 30 °C. This was almost the same as that observed under SCC, and β-carotenoid accumulation was more significant under R50W50 than that under W100 conditions, suggesting that red-light irradiation was suitable for β-carotenoid production. These results indicated that D3–1 is a good producer of not only lipids (TAGs) but also β-carotenoids. The green algae *Coelastrella* sp. can coproduce lipids and β-carotenoids at temperatures below 25 °C [13], [14], [15], [16]. The coproduction by D3–1 is advantageous for rapid growth (high yield of biomass) at 30 °C and a low cost in 0.2BG11 compared to other *Coelastrella* strains. Next, we further examined the production of biomass and β-carotenoids by D3–1/F3–6 cells grown in a liquid medium or on BG11/0.2BG11 plates under SCC, compared to that under BIC.

For short-term accumulation of β-carotenoids, further experiments were conducted under BIC at 30 °C (Supplementary Fig. S3), and the results are shown in Supplementary Table S2. We confirmed a significant β-carotenoid accumulation using 0.2BG11 liquid culture for only 5 days under BIC (Supplementary Fig. S3, panel A), which is the earliest known to date among *Coelastrella* studies (Table 1).

### 3.4 Pigment production under different solid-culture conditions

When green- and red-stage D3–1/F3–6 cells were inoculated onto the BG11/0.2BG11 plate, the cells significantly changed color (red/orange) under R50W50 irradiation, but were still green under W100 for 26 days, showing that red-light (660 nm wavelength) irradiation is important for β-carotenoid accumulation under SCC (Supplementary Fig. S3). The results also showed that both green- and red-stage D3–1/F3–6 cells can potentially be regrown and reused on the plates, resulting in larger amounts of β-carotenoid accumulation when BG11 medium was used rather than 0.2BG11. Because the cells grown on the plate were drier than those grown in the liquid culture, red-light irradiation was effective at 30 °C for a short period of 26 days for the accumulation of β-carotenoids under SCC. This is the first report indicating that regrowth and reuse are possible on the solid medium for *Coelastrella* cells.

### 3.5 Pigment compositions in cell extracts

Until now, composition analysis for pigments in cell extracts of *Coelastrella* has not been qualitatively and quantitatively analysed using TLC. Therefore, extracts prepared from the red (orange) stage cells grown on the SCC plate for 90 days were subjected to TLC analysis. The results are shown in Supplementary Fig. S4. Five pigments, βCar, Ec, Cx, Ax, and Chl *a* were recognised as the main components visualised on the TLC plate for both D3–1 and F3–6. This indicated that both D3–1 and F3–6 strains produced pigments with almost the same profile, as observed also for lipid production (Supplementary Fig. S4, Fig. 3).

Further analyses using not only TLC but also HPLC were performed for pigment compositions under the red (orange) cells grown under SCC and BIC (Supplementary Fig. S5) and the results are summarised in Supplementary Table S3. The results of these first attempts at compositional analysis using TLC and HPLC showed some differences in compositional ratios in both cases. For example, the composition ratios of Ec and Cx tended to be higher as a result of TLC analysis compared to HPLC, while those of axe were higher as a result of HPLC analysis compared to TLC (Fig. S5, Table S3). Therefore, in the present analysis, the compositional ratios of pigments were evaluated by averaging values obtained via TLC and HPLC, where the sum of the ratios for five major pigments (βCar, Ec, Cx, Ax, and Chl*a*) under BIC and SCC was taken as 100% (Tables S3, 1). Eventually, the average composition of βCar, Ec, Cx, Ax, and Chl *a* was calculated as 0.25, 30.0, 37.5, 31.5, and 0.71% (w/w), respectively, under SCC and as 0.56, 14.4, 41.1, 33.8, and 10.2% (w/w), respectively, under BIC. From the results, we interpreted that the red-stage D3–1 produced 16.04 mg *L*^−1^ *d*^−1^ (250 mg × 0.385/L/6d) of carotenoid extracts under BIC (Table 1). This value might indicate the superior carotenoid productivity of strain D3–1 compared to other *Coelastrella* strains.

We also elucidated the pigment composition of green-stage D3–1 cells compared to those of red (orange)-stage cells (Fig. S5, Table S3). Results showed that βCar, Ec, Cx, Ax, and Chl *a* were produced at 1.28, 14.0, 6.90, 16.9, and 60.9% (w/w), respectively, in the green extract from SCC (Fig. 3, panel A). This shows an apparent compositional change from abundant β-carotenoids in the red (orange) extract to chlorophylls in the green extract. Carotenes only consist of carbon and hydrogen as unsaturated hydrocarbons and give color to carrots, tomatoes, flamingos, salmon, shrimp/crab, and others. The biosynthesis pathway is as follows: Geranylgeranyl diphosphate (GGPP) → lycopene → βCar → Ec → Cx → Ax (Fig. 3). Since the D3–1 red (orange) and green extracts contained abundant Ec/Cx/axe and chlorophylls, their functions or effects on the cells were examined further.

### 3.6 Stress resistance of red/green cells

The stress resistance of the red/green cells was examined and the results are shown in Fig. 4. Red-stage cells showed higher resistance than green-stage cells (red frame) when plated (I) after decompression drying and (II) being cultured in the presence of a strong acid at pH 2. In contrast, green-stage cells showed higher resistance than red-stage cells (green frame) when plated (III) after oxidation with H_2_O_2_ and (IV) decompression drying. Although it is interesting to note that the results of (I) and (IV) were dependent more on being grown on a plate than in liquid culture; however, we could not elucidate the underlying reasons. The air around cells grown on a plate might exhibit stronger resistance to decompression drying stress than that around cultured cells. The reason why red cells grown in liquid culture showed resistance to a strong acid (II) might be the acquisition of antioxidant capacity related to carotenoid accumulation, as discussed in a later experiment (Fig. 5). For a similar reason, the antioxidant capacity of green cells with chlorophyll might confer resistance to oxidation with H_2_O_2_ (III) (Fig. 5). In contrast, both red and green cells were sensitive to NaClO in the range of 300–3,000 ppm, with a threshold of 250–300 ppm. This may be advantageous for the effective disinfection of the culture tank after outdoor cultivation of *Coelastrella* sp. D3–1. Because both red and green D3–1 cells can be frozen, thawed, recultivated, and stored for a long time. In addition, red/green D3–1 cells exhibited resistance to high-temperature stress at 40–60 °C which might depend on the cell structure or the quality and quantity of substances stored in the cell. As mentioned above, this is the first report examining the resistance of red/green *Coelastrella* sp. strain cells grown on plates or in liquid culture to environmental stress. In the future, it may provide valuable information for large-scale indoor or outdoor cultivation of not only D3–1/F3–6 cells but also other *Coelastrella* sp. strains.

### 3.7 Antioxidant capacities of red/green extracts

We tested the antioxidant capacities of the green- and red-stage extracts from D3–1 cells, and the results are shown in Fig. 5, where large values indicated high activity. Both green- and red-stage extracts from SCC showed significant antioxidant capacities depending on the concentration when Fx and βCar were used as controls. In this experiment, red-stage extracts prepared from *Haematococcus* sp. strain NIES-144 grown in BG11 (or CB) medium were also used as a positive control and the antioxidant properties were also confirmed (data not shown). The NIES-144 strain is a freshwater species of *Chlorophyta* from the family Haematococcaceae, and is an effective producer of axe, exhibiting a significant antioxidant capacity. The capacity of the green-stage D3–1 extract was approximately 3.5 [(80/58%) × (1,060/420 μg) = 3.54] folds higher than that of the red-stage D3–1 extract. These results indicated that both green- and red-stage extracts from the *Coelastrella* sp. D3–1 strain contained elements that acted as reducing agents and could be useful as antioxidant materials. This is the first report of both green- and red-stage extracts containing chlorophyll and β-carotenoid pigments showing apparent antioxidant capacities in microalgae.

### 3.8 Anti-inflammatory analysis for red/green extracts

In the assay for anti-inflammatory, small values indicated high activity. Results showed that both green and red extracts under SCC showed significant anti-inflammatory response via NO production induced by LPS (Fig. 6). IC50 values were 0.107 for the green-stage extracts and 0.274 for the red-stage extracts, suggesting that the anti-inflammatory effect of the green extract was approximately seven [(0.274/0.107) × (17.5/6.50) = 6.89] folds higher than that of the red extract. This result was also consistent with the antioxidant effect, showing that D3–1 extracts might be useful anti-inflammatory agents.

## 4 Conclusions

The novel green alga *Coelastrella* sp. strain D3–1 is a good co-producer of lipids and carotenoids at the red (orange) stage at 30 °C for only 5–6 days under specific BIC. The red/green-stage cells indicated stress resistance to pH 2–11, high temperatures of 40–60 °C, UV irradiation, drought, and H_2_O_2_ treatment under different culture conditions, thus exhibiting robust nature. Both red/green extracts showed antioxidant and anti-inflammatory properties, conferring new insights that they are potential materials for biorefineries.

## Declaration of Competing Interest

None

## Data Availability

No data was used for the research described in the article.

## Appendix Supplementary materials

Image, application 1
