# Supplementary material for: Coproduction of lipids and carotenoids by the novel green alga Coelastrella sp. depending on cultivation conditions
Source: Biotechnol Rep (Amst). 2022 Oct 24;37:e00769. doi: 10.1016/j.btre.2022.e00769 (PMC9843265; doi:10.1016/j.btre.2022.e00769)
Supplement: Supplementary file 1 [file mmc1.pdf]

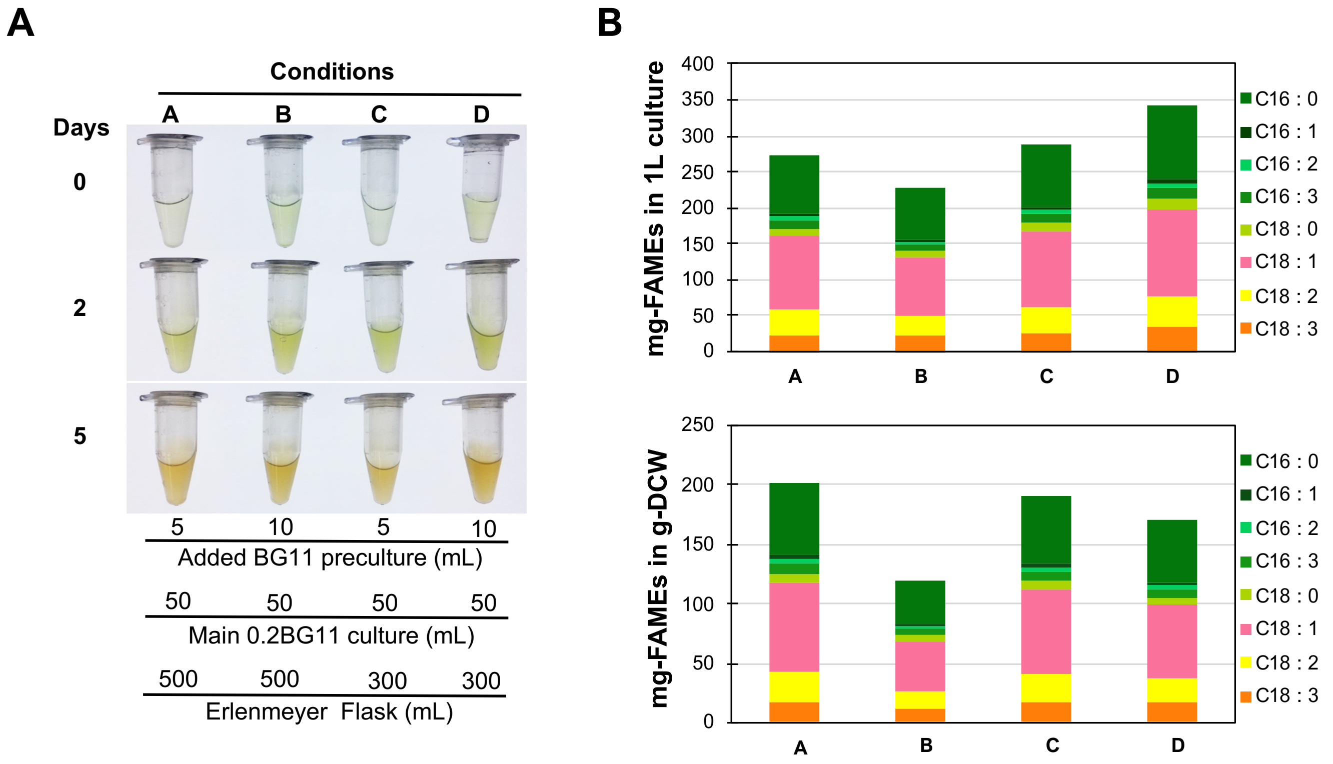


## Fig. S1. Lipid production in D3-1 cells in the 0.2BG11 medium

(**A**) Cultivation conditions. The D3-1 cells were cultivated under BICs for 5 days. Aliquots of the cell cultures are also shown. (**B**) FAMEs production. FAMEs extracted from the cells were analysed using GC/FID. Accumulated FAMEs were expressed per 1 L of cell culture and per DCW. Analyses were performed in independent duplicate experiments and the error margins were less than 7%.

**Table S1**

Lipid production in the D3-1 strain

| FAMEs | A | B | C | D |
|---|---|---|---|---|
| C16:0 | 29.7 | 30.6 | 29.8 | 30.3 |
| C16:1 | 1.89 | 1.78 | 1.87 | 1.76 |
| C16:2 | 1.78 | 1.62 | 1.83 | 1.57 |
| C16:3 | 4.39 | 4.56 | 4.27 | 4.68 |
| C18:0 | 3.78 | 3.75 | 4.02 | 3.67 |
| C18:1 | 37.2 | 35.5 | 37.0 | 35.5 |
| C18:2 | 12.5 | 12.6 | 12.7 | 12.7 |
| C18:3 | 8.75 | 9.57 | 8.51 | 9.82 |

FAMEs were analysed via GC/FID and the composition is shown as percentages in dry-cell weight (DCW), referring to Figure S1. Analyses were performed in independent duplicate experiments and the error margins were less than 7%. FAME, fatty acyl methyl ester.

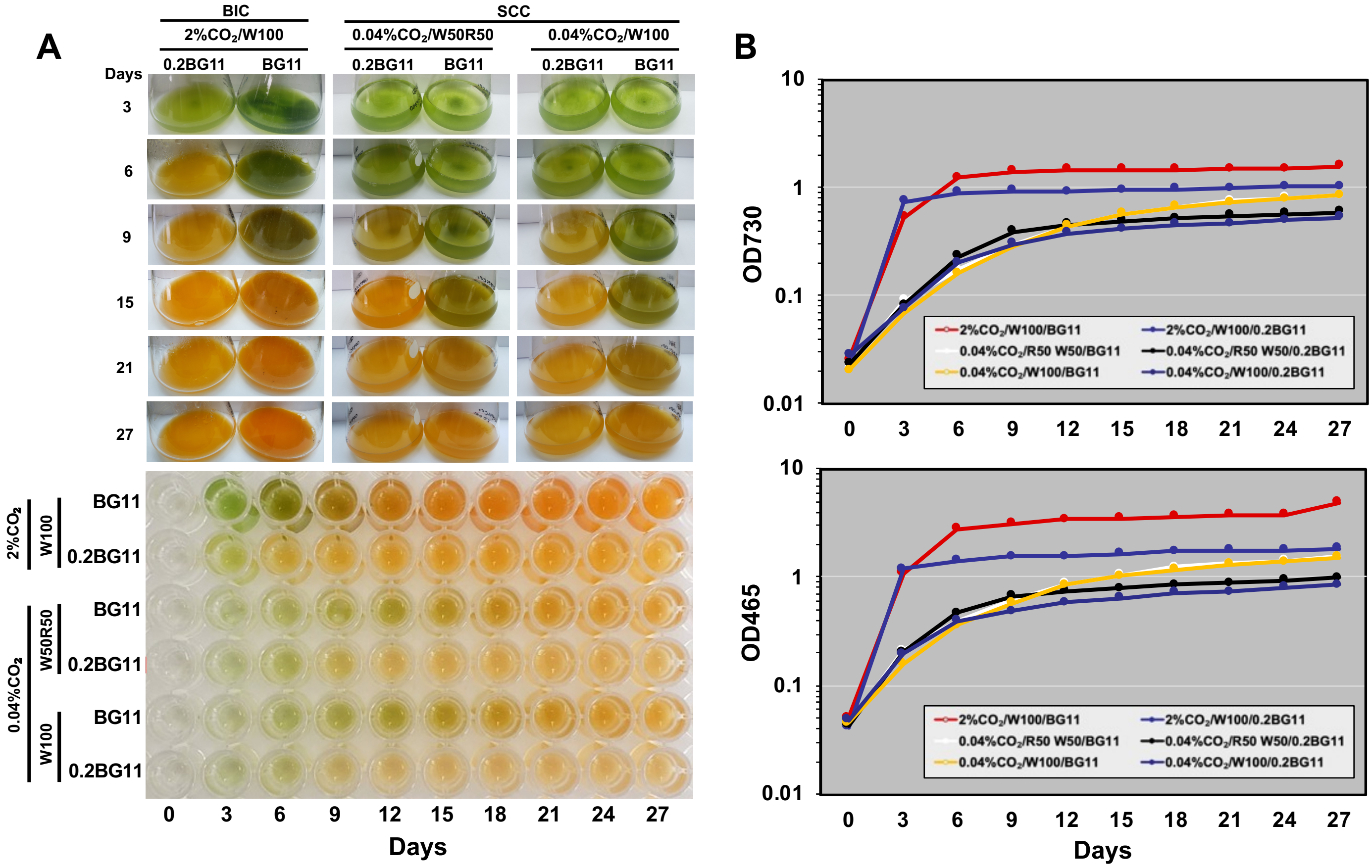


# Fig. S2. Liquid-culture conditions for pigment production

(**A**) Cultivation conditions and the colour of culture. The D3-1 cells were cultivated at 30°C under BIC or SCC for carotenoid (and lipid) accumulation (Top). Aliquots of the cell cultures in a 96-well plate are also shown for the colour observations from green to red (orange) stage (Bottom). W100, 100 μmol photons $m^{-2}$ $s^{-1}$ of white-light LED; W50R50, 50 + 50 μmol photons $m^{-2}$ $s^{-1}$ of white + red-light LED. (**B**) Culture turbidities were measured at $OD_{730}$ for cell growth (Top) or at $OD_{465}$ for β−carotenoid accumulation (Bottom).

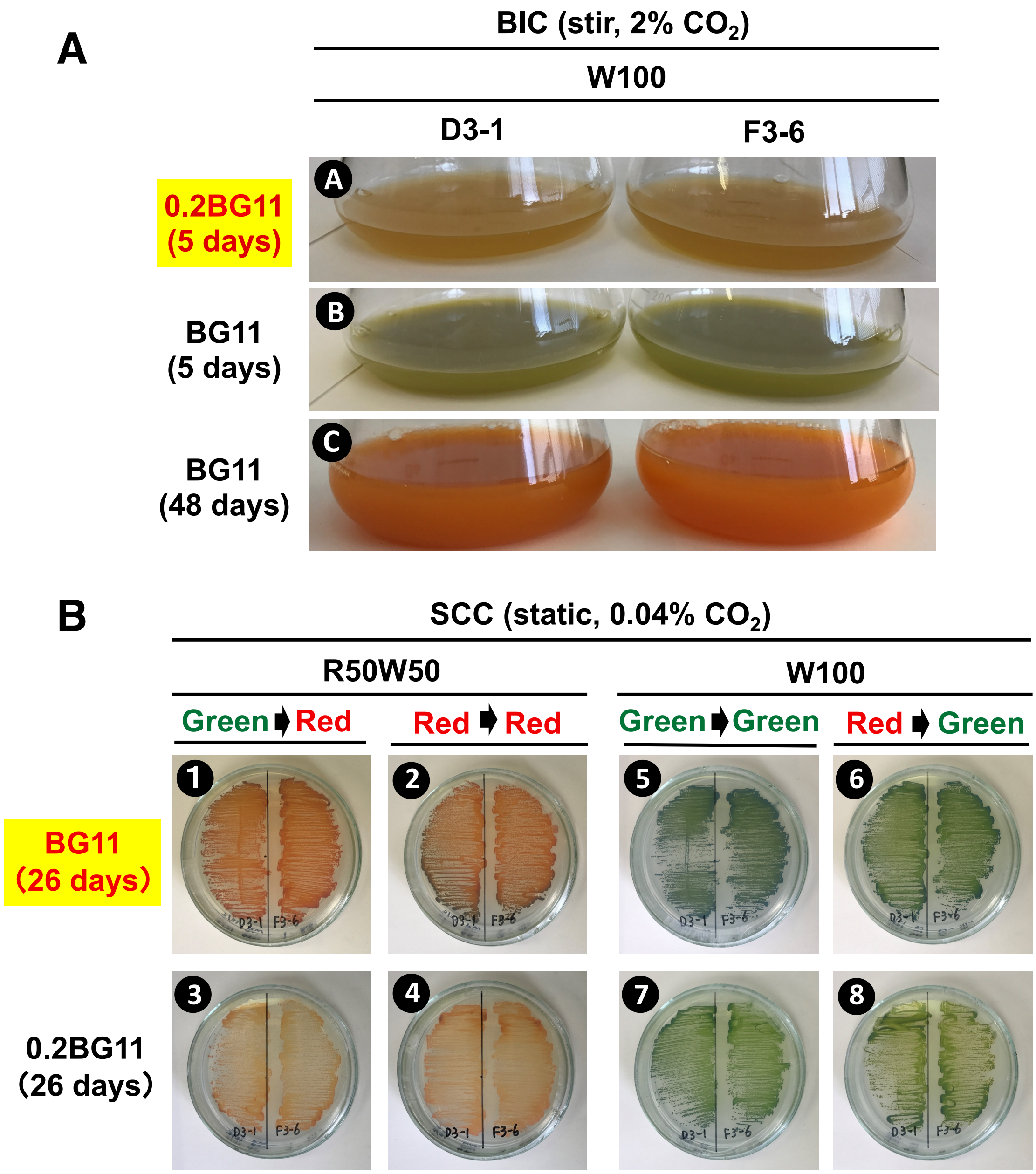


**Fig. S3. Pigment production depending on liquid/solid-culture conditions**

(**A**) The D3-1/F3-6 cells were cultivated for 5−48 days in 0.2BG11/BG11 liquid medium (left, D3-1; right, F3-6) at 30°C under liquid BICs (Figure S2). (**B**) Cultivation conditions and the color of cells. The D3-1/F3-6 (Figure S1) cells were cultivated for 26 days on plates (left, D3-1; right, F3-6) at 30°C under SCC. Approximately the same amounts of aliquots from the green-stage or red-stage D3-1/F3-6 cells were inoculated onto BG11/0.2BG11 plates as “Green→Red/ Green→Green” (plate numbers 1, 3/ 5, 7) or “Red→Red/ Red→Green” (plate numbers 2, 4/ 6, 8), respectively. W100, 100 μmol photons $m^{-2}$ $s^{-1}$ of white LED light; W50R50, 50 + 50 μmol photons $m^{-2}$ $s^{-1}$ of white+red LED light.

**Table S2**

Cultivation conditions for D3-1 and the cell state

| No. | Type | Culture | $\%CO_2$ | LED | Days | Cell color | Biomass |
|---|---|---|---|---|---|---|---|
| A | Liquid **BIC** | 0.2BG11 | 2.0 | W+W | 5 | Green → Red | Medium |
| B | | BG11 | 2.0 | W+W | 5 | Green → Green | High |
| C | | BG11 | 2.0 | W+W | 48 | Green → Red | High |
| 1 | Plate **SCC** | BG11 | 0.05 | R+W | 26 | Green → Red | High |
| 2 | | BG11 | 0.05 | R+W | 26 | Red → Green → Red | High |
| 3 | | 0.2BG11 | 0.05 | R+W | 26 | Green → Red | Low |
| 4 | | 0.2BG11 | 0.05 | R+W | 26 | Red → Green → Red | Low |
| 5 | | BG11 | 0.05 | W+W | 26 | Green → Green | High |
| 6 | | BG11 | 0.05 | W+W | 26 | Green → Green | High |
| 7 | | 0.2BG11 | 0.05 | W+W | 26 | Green → Green | Low |
| 8 | | 0.2BG11 | 0.05 | W+W | 26 | Green → Green | Low |

Cultivation conditions (30℃, $\%CO_2$, LED light as 100 µmol $m^{-2}$ $s^{-1}$) for D3-1 and the cell state are shown. This is summarised from the results shown in Figure S3. The numbers A–C or 1–8 correlate to those in Figure S3. R, red light; W, white light. BIC, basal induction conditions; SCC, standard cultivation conditions.

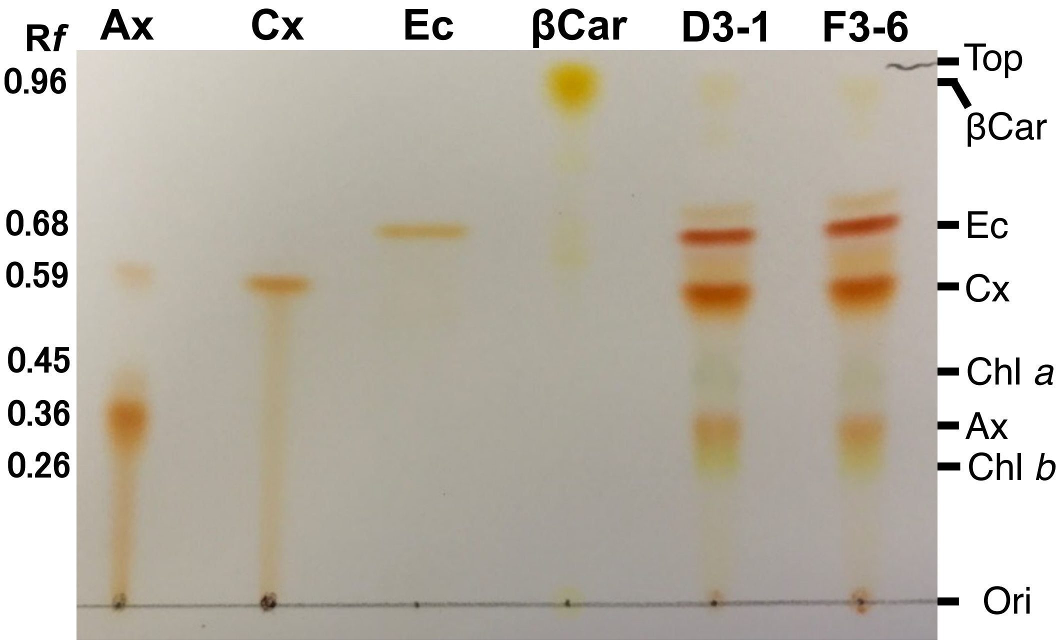


## Fig. S4. Pigment analysis using TLC

The sample extracts were prepared from the cells grown on the SCC plate for 90 days. Thin–layer chromatography (TLC) analysis of β-carotenoids and chlorophylls. Total extracts from the red (orange)-stage D3-1/F3-6 cells were prepared. A 4-μL aliquot of the extract was spotted on the origin (ori) of a silica gel plate. This plate was subjected to TLC with a developer eluent. Positions referring to respective pigments are shown as β-carotene (βCar), echinenone (Ec), canthaxanthin (Cx), astaxanthin (Ax), and chlorophyll *a* on the right. The R*f* values are shown on the left.

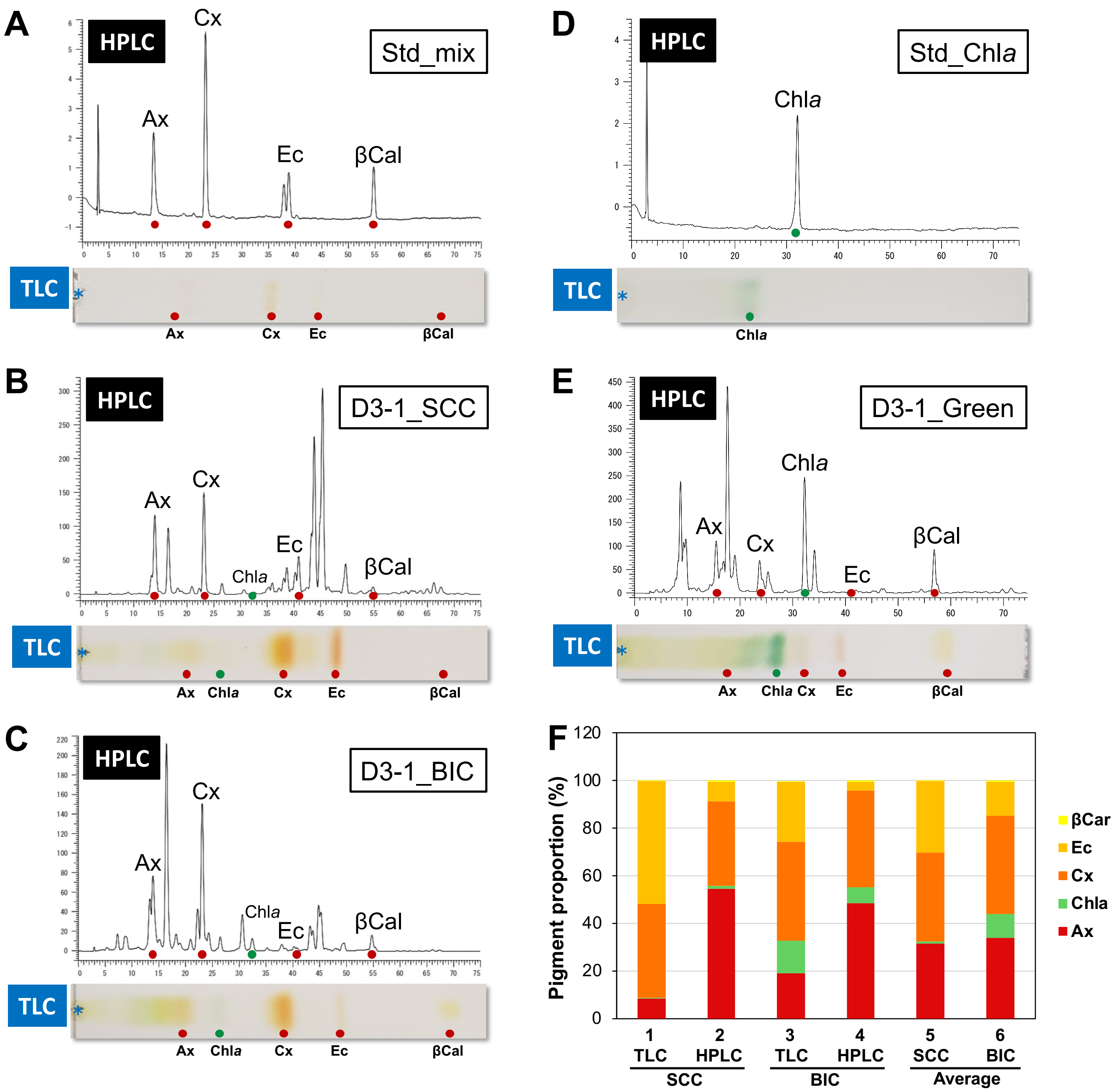


# Fig. S5. TLC and HPLC analyses for pigment production

The actual TLC and HPLC data are shown in panels A through E, respectively. (**A**) Standard mixture, (**B**) red extract from D3-1_SCC, (**C**) red extract from D3-1_BIC, standard mixture, (**D**) Chl *a* standard, and (**E**) green extract from D3-1_SCC. (**F**) A summarised graph for the proportion of the pigments obtained from the results of TLC and HPLC in which the numbers on bars accord to those in Table S3. Analyses were performed in independent triplicate experiments and the error margins were less than 5%. ●, positions for pigments; ∗, ori on a TLC plate.

# Table S3

Proportion of D3-1 pigments evaluated using TLC and HPLC

| | G-1 | G-2 | G-3 | 1 | 2 | 3 | 4 | 5 | 6 |
|---|---|---|---|---|---|---|---|---|---|
| **Phase** | **Green** | | | **Red** | | **Red** | | **Red** | |
| **Analysis[1)]** | **TLC[2)]** | **HPLC[3)]** | **Ave.[4)]** | **TLC[2)]** | | **HPLC[3)]** | | **Ave.[4)]** | |
| | | | | **SCC** | **BIC** | **SCC** | **BIC** | **SCC** | **BIC** |
| **βCal** | 0.29 | 2.26 | **1.28** | 0.10 | 0.46 | 0.39 | 0.66 | **0.25** | **0.56** |
| **Ec** | 22.7 | 5.25 | **14.0** | 51.9 | 25.1 | 8.27 | 3.63 | **30.0** | **14.4** |
| **Cx** | 13.0 | 0.80 | **6.90** | 39.5 | 41.7 | 35.5 | 40.5 | **37.5** | **41.1** |
| **Ax** | 17.4 | 16.4 | **16.9** | 8.43 | 19.0 | 54.5 | 48.5 | **31.5** | **33.8** |
| **Chl*a*** | 46.6 | 75.2 | **60.9** | 0.08 | 13.6 | 1.34 | 6.72 | **0.71** | **10.2** |
| **Total[5)]** | 100 | 100 | 100 | 100 | 100 | 100 | 100 | 100 | 100 |

[1)] Analyses were performed in independent triplicate experiments and the error margins were less than 5%.
[2)] Values (%) were evaluated by measuring signal intensities correlating to pigments on a TLC silica gel plate.
[3)] Values (%) were evaluated by measuring peak area values correlating to pigments in HPLC.
[4)] Values (%) are shown as averages (Ave.) from the respective results of TLC and HPLC.
[5)] Total = Four red pigments + Chl *a* = 100%.
